# Finding a Hadamard Matrix by Simulated Quantum Annealing

**DOI:** 10.3390/e20020141

**Published:** 2018-02-22

**Authors:** Andriyan Bayu Suksmono

**Affiliations:** Telecommunication Engineering Scientific and Research Group (TESRG), School of Electrical Engineering and Informatics and The Research Center on Information and Communication Technology (PPTIK-ITB), Institut Teknologi Bandung, Jl. Ganesha No.10, Bandung 40132, Indonesia; suksmono@stei.itb.ac.id

**Keywords:** quantum annealing, adiabatic quantum computing, hard problems, Hadamard matrix, binary optimization

## Abstract

Hard problems have recently become an important issue in computing. Various methods, including a heuristic approach that is inspired by physical phenomena, are being explored. In this paper, we propose the use of simulated quantum annealing (SQA) to find a Hadamard matrix, which is itself a hard problem. We reformulate the problem as an energy minimization of spin vectors connected by a complete graph. The computation is conducted based on a path-integral Monte-Carlo (PIMC) SQA of the spin vector system, with an applied transverse magnetic field whose strength is decreased over time. In the numerical experiments, the proposed method is employed to find low-order Hadamard matrices, including the ones that cannot be constructed trivially by the Sylvester method. The scaling property of the method and the measurement of residual energy after a sufficiently large number of iterations show that SQA outperforms simulated annealing (SA) in solving this hard problem.

## 1. Introduction

### 1.1. Background

Finding a solution to a hard problem is a challenging task in computing. Such a problem is characterized by its complexity, as it grows beyond the polynomial against the size of the input. A class of particularly important ones are NP (non-deterministic polynomial) problems, in which verifying a solution can be conducted in polynomial time, whereas finding the solution is of exponential order. Examples of such problems are, among others, the TSP (traveling salesman problem), SAT (Boolean satisfiability), graph coloring, graph isomorphism, and subset sums.

An interesting approach to the hard problems is a method inspired by physical phenomena, such as classical annealing (CA) or quantum annealing (QA). Both CA and QA are physical processes that obtain an ordered (physical) system from an unordered one, which can be done either thermally (as is the case in CA) or quantum-mechanically (as is the case in QA). To simulate the physical processes on a (classical/non-quantum) computer, numerical methods, such as MC (Monte Carlo) for CA and PIMC (path-integral Monte Carlo) for QA, have been developed. The algorithm or computational method inspired by classical/thermal annealing is called simulated annealing (SA), whereas the one based on quantum annealing is called simulated quantum annealing (SQA). Both of these methods make use of the methods in numerical CA or numerical QA. They encode the problem into a Hamiltonian of a spin system [1] and then evolve the system from a high energy state down to the ground state. The annealing process enables the system to avoid local minima trapping and therefore is capable of achieving a global optimum, which represents the best solution of the problem. The main difference between SA and SQA is in the evolution of the systems; whereas SA uses classical/thermal annealing, SQA employs quantum mechanism.

In SA [2,3,4], one starts the system in total randomness with regard to a high temperature state. The temperature is then lowered and the system is evolved, which causes the energy to decrease so that the system becomes increasingly ordered. To avoid local-optima trapping, a particular updating rule, such as the Metropolis [2], is applied. The rule allows the system to (sometimes) move to a higher energy state. Upon completion of the algorithm, the system achieves the ground state, at which point a solution is found.

In [5], Kadowaki and Nishimori introduced quantum fluctuations to replace the thermal fluctuations in SA to accelerate the convergence. They applied the method on an Ising model, where a transverse field plays the role of temperature in classical SA, enabling the system to achieve the ground state with greater probability. Santoro et al. [6] compared classical and quantum Monte Carlo annealing protocols on a two-dimensional Ising model. They found that the quantum Monte Carlo annealing is superior to classical annealing. In [7], Boixo et al. show experimental results on a  108 qubit *D-Wave One*, which is a kind of hardware implementation of QA. A strong correlation between D-Wave and SQA, compared to the device with classical annealing, was found, which indicates that the D-Wave performs quantum annealing. This result raised the important issue of whether QA actually outperforms SA [8]. Rønnow et al. [9] showed how quantum speedup should be defined and measured. In an experiment with random spin glass instances on 503 qubits of *D-Wave Two*, they did not find any evidence of such speedup.

Regardless of these issues, different results have been achieved via SQA. Isakov [10] performed quantum Monte Carlo (QMC) simulations and found that the QMC tunneling rate displayed scaled according to system size. He also found quadratic speedup in QMC simulations when, instead of periodic conditions, open boundary conditions were employed. In [11], Mazzola et al. demonstrated that QMC simulations can recover the scaling of ground-state tunneling rates, which validates QA in terms of solving combinatorial problems.

Some classes of hard problems, including ones with exponential or combinatorial complexity, have been a subject of interest in SQA research. Martonak et al. [12] introduced an application of SQA to solve the TSP problem. They found that a PIMC algorithm was more efficient than SA in terms of finding an approximately minimal tour in a given graph. SQA has also been used to successfully address other hard problems related to graphs, such as graph coloring [13] and graph isomorphism [14].

In this paper, we propose SQA as a mean to find a Hadamard matrix (H-matrix). Previously, in [15], we successfully employed SA to perform a similar task, in which low-order H-matrices were found. Compared to existing H-matrix construction methods, an SA-based method is more general in terms of its capability of finding (or constructing probabilistically) an m=4k order H-matrix, without any restriction on the property of the order *m*, whereas the Sylvester method requires $m=2^{n}$, where *k* and *n* are positive integers. This paper extends this classical SA method to its quantum version, where PIMC based on Suzuki–Trotter formulation [16,17] is employed to simulate the quantum process.

### 1.2. Finding A Hadamard Matrix

A Hadamard matrix, or H-matrix, is an orthogonal binary {±1} matrix of size 4k×4k, where *k* is a positive integer. This matrix was discovered by J. J. Sylvester [18] in 1867 and then studied more extensively by J. Hadamard [19] during his investigation of the maximal determinant problem. The orthogonal property makes the H-matrix popular in applied areas, such as information coding and signal transform. In the 1960s and 1970s, Hadamard code was used in space exploration for information transmission [20,21]. In a recent technological case, CDMA (code-division multiple access), which is widely used in cellular mobile phone systems, employs Walsh–Hadamard signals to reduce interference between its users [22,23].

One of the most important issues in the theory of H-matrix is its existence. Any $2^{l}$ order H-matrix with *l* a positive integer can be constructed using Sylvester’s method. Furthermore, if there is an *m* order H-matrix, m=4k can be shown for a positive integer *k*. On the other hand, no one yet knows if there is always a 4k order H-matrix [20,21]. The latter case is formulated as the Hadamard matrix conjecture. Up to this writing, the smallest unknown 4k order H-matrix is 668.

Various reconstruction methods have been proposed [24,25,26,27,28,29]. Nevertheless, these methods force the order *m* to follow a particular rule. In [15], a general m=4k order algorithm employing SA is proposed. The method works on a special H-matrix called a seminormalized Hadamard (SH) matrix, in which the first column is a 4k order unity vector ${\vec{v}}_{0}={\left(1,\cdots ,1\right)}^{T}$, and the rest are 4k order SH vectors ${\vec{v}}_{i}\in V$.

A brute-force method needs to verify all $N_{B}$ of the 4k order binary matrix to find an H-matrix, where $N_{B}\left(4k\right)=2^{16k^{2}}$ [15]. Let all matrices constructed where ${\vec{v}}_{0}$ is the first column and a combination of ${\vec{v}}_{i}\in V$ constitutes the remaining (4k−1) columns be called quasi-SH (QSH) matrices. Since there are $N_{V}=C\left(4k,2k\right)$ SH vectors, there are about $N_{QU}\left(4k\right)\approx {\left(\frac{2^{4k}}{8k^{3/2}}\right)}^{4k}$ unique QSH-matrices. Although the number has been greatly reduced compared to $N_{B}$, exhaustive checking still requires a great amount of computational resources. The SA method proposed in [15] is capable of finding a few low-order SH matrices in a more reasonable time.

Following the convention in our previous paper [15], the role of the spin, i.e., its ±1 eigenvalues, is replaced by SH spin vectors ${\vec{v}}_{i}\in V$. To find a 4k order SH-matrix, one needs (4k−1) fully connected SH spin vectors, which initially are set randomly. With a defined energy $E(\vec{Q})$, the SH spin vectors are randomly changed in accordance with conditions whereby a transition into another SH spin vector is allowed but a transition into a non-SH-spin-vector is forbidden.

## 2. Methods

### 2.1. Simulated Quantum Annealing

The Hamiltonian of an Ising system with spin configuration $\left\{{\hat{\sigma }}_{k}\right\}$, where k∈K={1,2,⋯,i,j,⋯} is the set of the lattice’s indices, can be expressed as

(1) $$\hat{H}=-\sum_{i\neq j}J_{ij}{\hat{\sigma }}_{i}^{z}{\hat{\sigma }}_{j}^{z}-\sum_{i}h_{i}{\hat{\sigma }}_{i}^{z}$$

where $J_{ij}$ is a coupling constant/strength between a spin at site *i* with a spin at site *j*, $h_{j}$ is the magnetic strength at site *j*, and $\left\{{\hat{\sigma }}_{i}^{z},{\hat{\sigma }}_{i}^{x}\right\}$ are Pauli’s matrices at site *i*. In SQA, quantum fluctuation is elaborated by introducing a transverse magnetic field Γ. The Hamiltonian of the system takes the following form [5]:

(2) $${\hat{H}}_{QA}=-\sum_{i\neq j}J_{ij}{\hat{\sigma }}_{i}^{z}{\hat{\sigma }}_{j}^{z}-\sum_{i}h_{i}{\hat{\sigma }}_{i}^{z}-\Gamma \sum_{i}{\hat{\sigma }}_{i}^{x}.$$

In Equation (2), the transverse field is changed (reduced) over time, i.e., Γ≡Γ(t). On the right hand side of the equation, the first two terms corresponds to potential energy ${\hat{H}}_{pot}$, while the third one is the Hamiltonian introduced by the transverse field, which is related to kinetic energy ${\hat{H}}_{kin}$; i.e, we can define

(3) $${\hat{H}}_{pot}\equiv -\sum_{i\neq j}J_{ij}{\hat{\sigma }}_{i}^{z}{\hat{\sigma }}_{j}^{z}-\sum_{i}h_{i}{\hat{\sigma }}_{i}^{z}$$

(4) $${\hat{H}}_{kin}\equiv -\Gamma \sum_{i}{\hat{\sigma }}_{i}^{x}.$$

In general, ${\hat{H}}_{pot}$ and ${\hat{H}}_{kin}$ do not commute, so $[{\hat{H}}_{pot},{\hat{H}}_{kin}]\neq 0$. Denoting the Hamiltonian of the potential as a function of spin configurations ${\hat{H}}_{pot}\equiv \hat{H}\left(\{{\hat{\sigma }}_{i}^{z}\}\right)$, we can also express Equation (2) in a more general form as follows:

(5) $${\hat{H}}_{QA}=-\hat{H}\left(\{{\hat{\sigma }}_{i}^{z}\}\right)-\Gamma \sum_{i}{\hat{\sigma }}_{i}^{x}.$$

To simulate a quantum system described by Equation (5) using the classical method, we have to formulate PIMC by introducing imaginary time. It can be then approximated by the Suzuki–Trotter transform by adding one dimension in the imaginary time direction, which, for (P×N) degrees of freedom, takes the following form [13,30]:

(6) $$H_{ST}=\frac{1}{P}\sum_{p=1}^{P}H_{pot}\left(\{S_{i,p}\}\right)-J_{\Gamma }\left(\sum_{p=1}^{P-1}\sum_{i}^{N}S_{i,p}S_{i,p+1}+\sum_{j}^{N}S_{j,1}S_{j,p}\right)$$

where *N* is the number of spins in the lattice, *P* is the number of Trotter’s replicas, $S_{i}=\pm 1$ are the eigenvalues of the spin matrices, and

(7) $$J_{\Gamma }=-\frac{PT}{2}\ln\tanh\left(\frac{\Gamma }{PT}\right)>0$$

is the nearest-neighbor coupling of the transverse magnetic field [30].

### 2.2. SQA Formulation of the SH Spin Vector

Similar to the previous paper [15], we employ a seminormalized Hadamard spin vector, abbreviated here as an SH spin vector, instead of an ordinary spin. In a 4k order SH spin vector, for a given positive integer *k*, 2k spins are −1 and another 2k spins are +1. Therefore, an SH spin vector transition is allowed only if these balance numbers are conserved; otherwise, such a transition is forbidden. We also treat the SH spin vector as a single entity, even though it consists of 4k spins, and is denoted as ${\vec{v}}_{i}\in V$, where *V* is the set of all 4k-order SH vectors. We formulate the energy of a particular configuration of spin vectors $\left\{{\vec{v}}_{i}\right\}$ as follows:

(8) $$E\left(\left\{{\vec{v}}_{i}\right\}\right)=\left|\sum_{i\neq j}{\vec{v}}_{i}\cdot {\vec{v}}_{j}+\sum_{i}\vec{1}\cdot {\vec{v}}_{i}\right|-16k^{2}$$

where ${\vec{v}}_{i}\cdot {\vec{v}}_{j}$ denotes the inner product of the vector ${\vec{v}}_{i}$ with ${\vec{v}}_{j}$.

**Figure 1:**
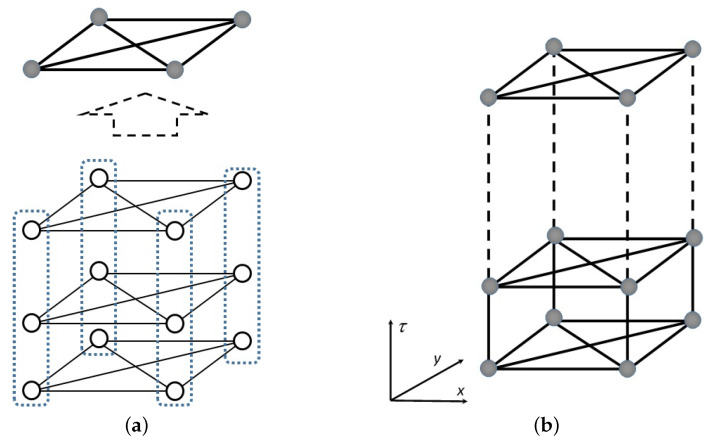
Connection diagrams of the spins and spin vectors. We consider a four-order SH vector in this example: (**a**) four SH spins are connected by a complete graph $K_{4}$, and each column is then grouped into a single SH spin vector; (**b**) an extension of fully connected SH spin vectors into a Trotter dimension (imaginary time) τ.

Figure 1 shows an Ising system with four SH spin vectors with an additional Trotter’s dimension. In the lower part of Figure 1a, each circle represents a binary spin, whereas the solid line represents the connection among the spins. Interacting spin *i* with binary variable $S_{i}$ and spin *j* with binary variable $S_{j}$ contributes the term $J_{ij}S_{i}S_{j}$ to the Hamiltonian. For a 4k order case, every 4k non-connected spins are grouped into one SH vector ${\vec{v}}_{i}$, which is illustrated as a dashed line. To simplify the diagram, each SH vector is represented by a filled circle; thus, we obtain the upper part of Figure 1a, which is called a slice or a replica. In the PIMC, the slice is replicated *P*-times, and these slices are arranged as layers in imaginary time. Each neighboring SH vector in a replica, i.e., ${\vec{v}}_{i,p}$ with ${\vec{v}}_{i,p-1}$ and ${\vec{v}}_{i,p}$ with ${\vec{v}}_{i,p+1}$, interacts. The extension (in imaginary time) is illustrated in Figure 1b. The Hamiltonian in Equation (6) becomes a Hamiltonian of an SH vector spin system $H_{QV}$ that can be rewritten as follows:

(9) $$H_{QV}=\frac{1}{P}\sum_{p=1}^{P}H_{pot}\left(\{{\vec{v}}_{i,p}\}\right)-J_{\Gamma }\left(\sum_{p=1}^{P-1}\sum_{i}{\vec{v}}_{i,p}\cdot {\vec{v}}_{i,p+1}+\sum_{i}{\vec{v}}_{i,1}\cdot {\vec{v}}_{i,p}\right)$$

where $J_{\Gamma }\equiv J_{\Gamma }\left(t\right)$ and $H_{pot}\left(\{{\vec{v}}_{i,p}\}\right)$ represent complete-graph connections among the SH spin vectors, similar to Equation (8), which is given by

(10) $$H_{pot}\left(\{{\vec{v}}_{i,p}\}\right)=\left|\sum_{i\neq j}{\vec{v}}_{i,p}\cdot {\vec{v}}_{j,p}+\sum_{i}\vec{1}\cdot {\vec{v}}_{i,p}\right|-16k^{2}.$$

The evolution of $H_{QV}$ in Equation (9) leads to the solution to the H-matrix search problem.

We will now formulate the SQA method for finding the H-matrix into an algorithm, which is displayed as pseudo-code in Algorithm 1. It takes the matrix order, the number of replicas, the initial temperature, the initial value of Γ, and the amount of iterations and sub-iterations as inputs. This algorithm yields either an SH-matrix or a QSH-matrix that has more orthogonal column vectors than the initial one. The algorithm starts with a random initialization of replicas with QSH-matrices, which are (4k−1) sets of SH vectors, and then calculates its initial energy. Following the schedule of a linear transverse field, a trial transition is performed for each replica. The acceptance and rejection of the transition is based on the Metropolis criterion. The iteration will be stopped when either the number of maximum iterations is reached or an SH-matrix is found.

**Algorithm 1** Finding an H-Matrix via Simulated Quantum Annealing
1:**Input:** Order of SH-matrix 4k, number of replicas P, $T_{0}$, $\Gamma_{0}$, MaxIter, SubIter.2:**Output:** A 4k-order SH-matrix ${\vec{H}}_{F}$ or a partially orthogonal matrix $\vec{Q}$.3:Initialize $T=T_{0}$, $\Gamma =\Gamma_{0}$4:Initialize all-replicas R with randomly generated QSH-matrix: $R\leftarrow \{{\vec{Q}}_{1},...,{\vec{Q}}_{P}\}$5:idx←06:${\vec{H}}_{F}\leftarrow \vec{0}$7:*FLAG* ←08:**while** (idx<MaxIter) *or* (*FLAG* ==0) **do**9:     Calculate $J_{\Gamma }\left(idx;\Gamma ,P,T\right)$10:    Calculate current all-replicas energy: $E_{rep}=H_{QV}\left(R,J_{\Gamma }\right)$11:    r←012:    **while** r<P **do**13:        Select a replica at position *r*: ${\vec{Q}}_{r}$14:        Calculate potential energy of the replica: $E_{pot}=H_{pot}\left({\vec{Q}}_{r}\right)$15:        **if** $E_{pot}>0$ **then**16:           m←017:           **while** (m<SubIter) and (*FLAG* ==0) **do**18:               Flip SH spin vector randomly: ${\vec{Q}}_{r}\to {\vec{Q}}_{r}^{\prime }$19:               Calculate energy of the updated replica: $E_{pot1}=H_{pot}\left({\vec{Q}}_{r}^{\prime }\right)$20:               **if** $E_{pot1}$==0 **then**21:                   $E_{pot}\leftarrow E_{pot1}$22:                   ${\vec{H}}_{F}\leftarrow {\vec{Q}}_{r}^{\prime }$23:                   *FLAG* ←124:                   r←P25:               **else**26:                   Update all-replicas: $R\to R^{\prime }$27:                   Calculate energy of updated all-replicas $E_{rep1}\leftarrow H_{QV}\left(R^{\prime },J_{\Gamma }\right)$28:                   $\Delta E_{rep}\leftarrow E_{rep1}-E_{rep}$29:                   $\Delta E_{pot}\leftarrow E_{pot1}-E_{pot}$30:                   Perform a transition if allowed (Metropolis update rule):31:                   **if** $\left(\Delta E_{pot}<0\right)$ **or** ($\Delta E_{rep}<0$) **or** ($e^{-\frac{\Delta E_{rep}}{T}}>rand$) **then**32:                       Accept the transition: $R\leftarrow R^{\prime }$, $E_{rep}\leftarrow E_{rep1}$33:                   **end if**34:               **end if**35:               m←m+136:           **end while**37:        **else**38:           ${\vec{H}}_{F}\leftarrow {\vec{Q}}_{r}$39:           *FLAG* ←140:           r←P41:        **end if**42:        r←r+143:    **end while**44:**end while**

## 3. Numerical Experiments and Analysis

### 3.1. Finding a 12-Order SH-Matrix Using SQA

We have performed numerical experiments to find low-order H-matrices. Here we present results for the H-matrix of order 12 for detailed analysis, since it is the lowest-order H-matrix that cannot be constructed by the Sylvester method. Initially, all of the slices (replica) were filled with randomly generated ${\vec{v}}_{i}\in V$. Note that there are two nested iterations in Algorithm 1. The first one is an iteration of all replicas with the maximum number set to k·M×M, where M=12 is the H-order. The second one is an iteration of flipping within a slice of a replica, whose number is c·M, *c* can be any small number.

**Figure 2:**
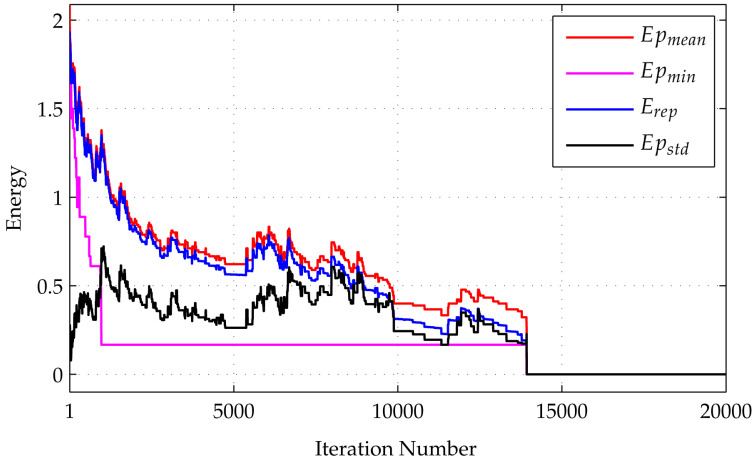
Energy evolution during the SQA algorithm runs to find an SH-matrix of order 12. Four curves are drawn in the graph, which are the mean potential energy $Ep_{mean}$, the minimum potential energy $Ep_{min}$, the replica energy $E_{rep}$, and the deviation standard of the potential energy $Ep_{std}$. When $Ep_{min}$ equals zero, the iteration is stopped since an SH-matrix has been found. The $Ep_{std}$ curve indicates high variation in the configuration of replicas at the initial stage, which is then reduced in later stages.

The energy evolution during the iteration is shown in Figure 2. The figure shows curves of replica energy $E_{rep}$, mean potential energy $Ep_{mean}$, and minimum potential energy $Ep_{min}$. The replica energy is defined similarly to Equation (9), i.e., $E_{rep}\equiv H_{QV}$, whereas the potential energy is given by Equation (10) $E_{pot}\equiv H_{pot}$. The mean and minimum values have been taken across the replicas. Based on the figure, both $Ep_{mean}$ and $E_{rep}$ fluctuate over time, but they tend to decrease. The minimum energy of a lattice in the replica $Ep_{min}$ also tends to decrease. When $Ep_{min}=0$, the H-matrix is found.

**Figure 3:**
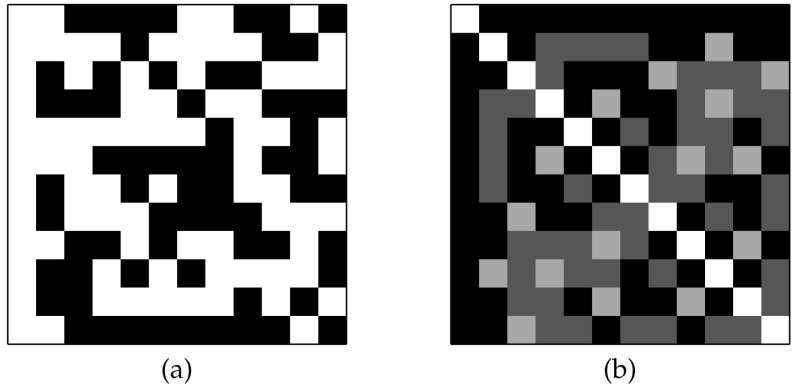
The initial state of the found H-matrix: (**a**) The QSH-matrix, white squares indicate +1, black squares indicate −1. (**b**) Orthogonality indicator, gray squares show the non-orthogonality condition of related pair of vectors.

**Figure 4:**
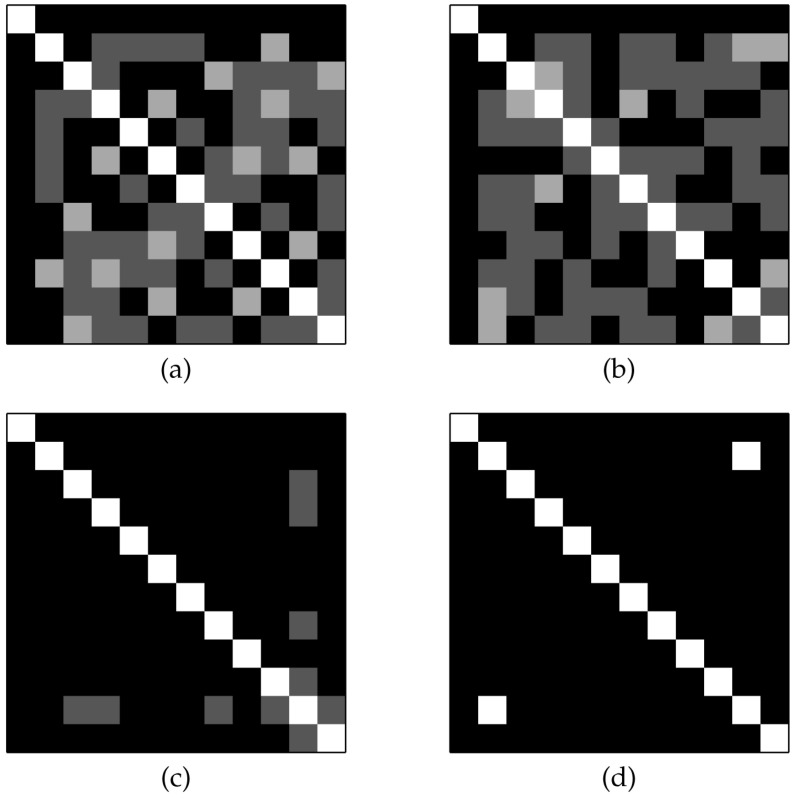
Indicator matrices of the replica content: (**a**) the first replica at the initial stage; (**b**) the last replica at the initial stage; (**c**) the first replica at the final stage, and (**d**) the last replica at the final stage. The matrices at the initial stages show most of the vectors as non-orthogonal, whereas those at the final stages show most of the vectors as orthogonal.

**Figure 5:**
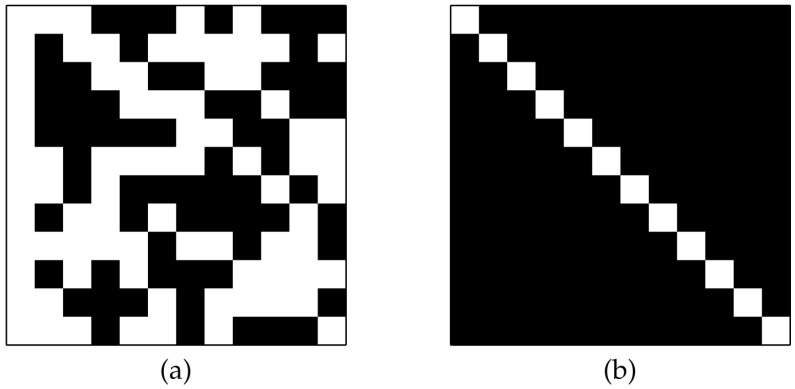
Final results: (**a**) the found H-matrix and (**b**) its orthogonality indicator. The diagonal form of the indicator matrix indicates that all of the column vectors are now orthogonal.

The degree of orthogonality of the matrix $\vec{Q}$ is displayed by the indicator matrix $\vec{D}\equiv {\vec{Q}}^{T}\vec{Q}$. Figure 3 shows the initial QSH-matrix and its related indicator matrix. We also show the initial and final indicators for the first and last slices of the replica in Figure 4. It is expected that all of the QSH-matrices become more orthogonal, indicated by a lower number of zeros in off-diagonal entries. The last figure showing the last slice of the replica condition after the iterations are completed clearly show this case. The found H-matrix is shown in the left part of Figure 5, with its corresponding indicator shown on the right, which is a diagonal matrix.

### 3.2. The Number of The Replicas and Convergence Issue

**Figure 6:**
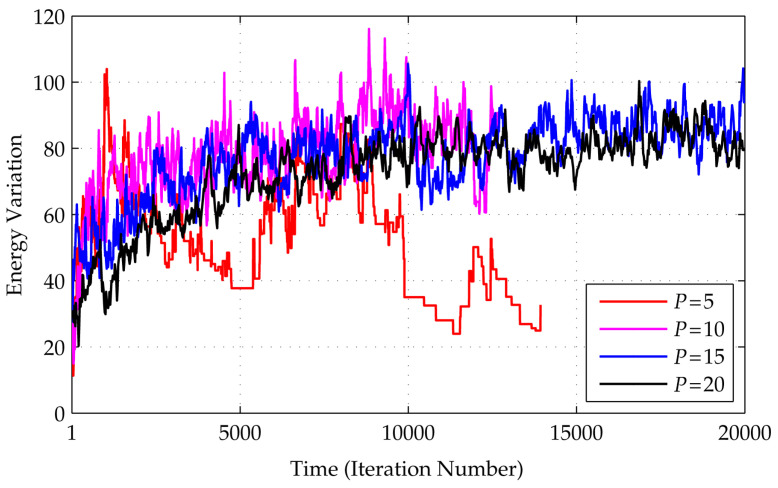
The effect of the replica number *P* in the algorithm: although ideally a large *P* is desired, it also needs to be adjusted to the problem. Variation in replica energy (in terms of deviation standards of the energy across the replicas) when searching for an H-matrix is shown. The numbers of replicas P=10,15,20 yield large variations up to the end of the iteration, whereas P=5 yields a better result with steady values at the end. In all of these cases, for the construction of a 12-order H-matrix, the total maximum iteration is set to 20,000, consisting of a global iteration count of 20,000 for each *P*.

In theory, the number of Trotter’s replicas *P* should be as large as possible. However, in practice, we should also consider the convergence issue when a running time restriction (iteration number) is given. As explained in [13,30], replicas provide diversity of solutions; a greater *P* selects the best solution with minimum energy. On the other hand, the replicas are not merely running Monte Carlo on several replicas; the interactions between replicas $J_{\Gamma }\left(t\right)$ also define their behavior, i.e., a large value of Γ at the initial stage implies a low value of $J_{\Gamma }$, which loosens the connections, and the interactions then become independent. A low Γ value at the end of an iteration implies a high $J_{\Gamma }$ value, which tightens the replica connections such that they become similar. To measure these variations, we used a simple deviation standard of energy across the replicas. Figure 6 shows the curves of variation of the energy evolution for P=5,10,15, and 20 in finding a 12-order H-matrix.

Since initially the replicas were set randomly, they will have almost identical energy, so variation in the energy will be very low. In later iterations, the value will increase as a new configuration is explored, and this will be followed by a decrease, which indicates that the replicas have become homogeneous. This cycle of increasing–decreasing energy should be observed if *P* is chosen properly with respect to the dimension of the problem (H-order) and a sufficient number of iterations. When *P* is too small, the system will perform akin to classical SA, whereas a *P* that is too large will cause the system to fail. The figure shows that, for a given number of maximum iterations 20,000, the number of replicas P=5 is the most suitable; anything higher is too high. This also shows that frequent updates on a limited number of replicas, compared to less frequent updates on a larger number of replicas, better achieve convergence.

### 3.3. Performance Comparison: SQA vs. SA

To compare performances, in the first experiment, we measured the residual error of both algorithms. Since the ground state is achieved when the matrix becomes orthogonal, in which case Equation (10) will equal zero, the residual error ϵ will be defined as the minimum $H_{pot}$ over all of the replicas, i.e., $\epsilon =\min\left(H_{pot}\right)$. We have chosen the order of the H-matrix to be sufficiently large so that we will still have a residual error at the end of the execution of the algorithm, i.e., so that the H-matrix is not found. We considered order M=28 to be sufficient for this purpose, where we actually have $28^{3}=$ 21,952 spins. We also chose a Trotter slice of P=5 and plotted the curve for iterations 50 up to 5,000,000.

**Figure 7:**
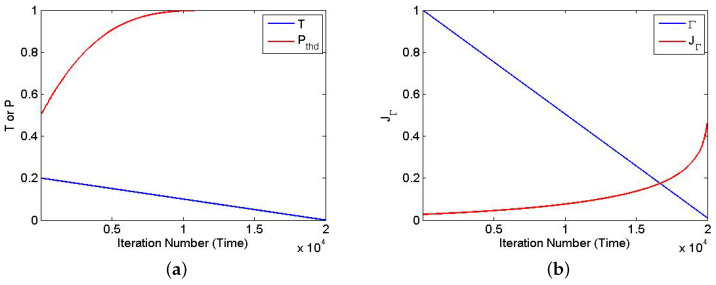
The annealing schedules in SA and SQA: (**a**) Linear temperature schedule and corresponding threshold schedule in SA. (**b**) Linear transverse-field Γ(t) and corresponding $J_{\Gamma }\left(t\right)$ in SQA.

Following [30], the annealing schedule was linear; i.e., the temperature *T* was reduced linearly in SA, and was the transverse magnetic strength Γ. Even though *T* is reduced linearly, the threshold probability $P_{thresh}$ will change exponentially. By using the function

(11) $$P_{thresh}\left(t\right)=1-\frac{1}{2}e^{\frac{-1}{T(t)}}$$

the threshold will start a bit higher than 0.5, which asymptotically approaches 1.0 at the end of iteration time *t*. Figure 7 shows the curve of T(t), $P_{thresh}\left(t\right)$, Γ(t), and $J_{\Gamma }\left(t\right)$.

**Figure 8:**
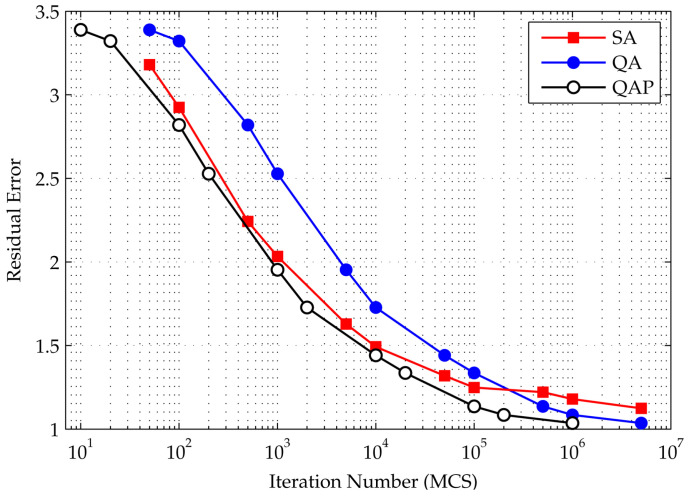
Residual energy left by the SA and SQA algorithms. The QAP curve shows when the horizontal axis accounts for the MCS (the Monte Carlo step); i.e., the number of iterations in the SQA curve is divided by the number of slices *P*. The figure shows that SQA outperform SA in finding an H-matrix. Even when the number of steps is counted without the MCS, SQA eventually outperforms SA at higher iterations, demonstrated by the steeper slope of the SQA performance curve, compared to SA.

The experiments were repeated 10 times for each case. The averages of residual errors for each iteration numbers are plotted in Figure 8 for both SA and SQA.

The figure shows that, although initially the residual error of SQA is larger than SA, the slope is steeper. With a higher number of iterations, which in this case is around 100,000, SQA is superior. Considering that SQA shows the least amount of error among the replica slices, it seems that variation in the replica is an ideal solution. In SA, once a solution is selected, the change in spin configuration will be less significant by the time the system reaches a lower energy state. Therefore, in terms of finding an H-matrix, SQA is superior to SA.

**Figure 9:**
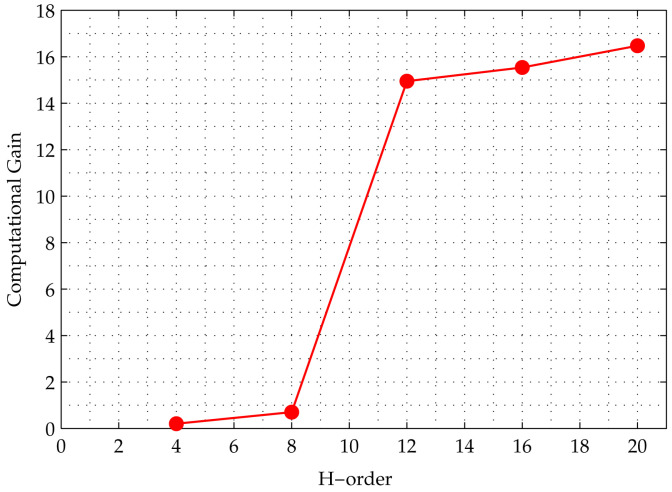
Curve of computational gain, which is the ratio of the number of SA iterations to the number of SQA iterations needed for the algorithm to achieve 50 percent of its initial residual error. The horizontal axis represent the problem size, which is the order of the H-matrix. The figure shows that the gain grows non-linearly with problem size, indicating that SQA outperforms SA.

In the second experiment, both SA and SQA were applied to matrices with an increasing size (order). Figure 9 shows a graph of computational gain, which is defined as the ratio of the number of SA iterations to the number of SQA iterations needed to achieve 50 percent of the residual energy of the initial mean energy of all replicas. The horizontal axis shows the order of the H-matrix, from 4 to 20, whereas the vertical axis shows the computational gain. The gain grows with the order of the H-matrix, which shows that speedup increases with problem size. Based on this curve, we observe that SQA outperforms SA for the Hadamard search problem.

## 4. Conclusions

We here propose a new method of finding an H-matrix based on SQA. We have formulated the method into an algorithm, which has been implemented, tested, and analyzed. Low-order H-matrices, including one of order 12 that cannot be constructed via the Sylvester method, were found. We have also discussed the advantages of the method over classical SA. Measurements of the residual error and the relative running time on an increasing order of H-matrices indicate that SQA is superior to SA in solving the Hadamard search problem.
